# Genome-Wide Association Study Identifies Novel Pharmacogenomic Loci For Therapeutic Response to Montelukast in Asthma

**DOI:** 10.1371/journal.pone.0129385

**Published:** 2015-06-17

**Authors:** Amber Dahlin, Augusto Litonjua, John J. Lima, Mayumi Tamari, Michiaki Kubo, Charles G. Irvin, Stephen P. Peters, Kelan G. Tantisira

**Affiliations:** 1 Channing Division of Network Medicine, Brigham & Women's Hospital and Harvard Medical School, Boston, MA, United States of America; 2 Division of Pulmonary and Critical Care Medicine, Brigham and Women's Hospital and Harvard Medical School, Boston, MA, United States of America; 3 Nemours Children’s Clinic, Jacksonville, FL, United States of America; 4 Center for Integrative Medical Sciences, RIKEN, Yokohama, Japan; 5 University of Vermont, Burlington, VT, United States of America; 6 Wake Forest School of Medicine, Winston-Salem, NC, United States of America; National Cancer Institute, National Institutes of Health, UNITED STATES

## Abstract

**Background:**

Genome-wide association study (GWAS) is a powerful tool to identify novel pharmacogenetic single nucleotide polymorphisms (SNPs). Leukotriene receptor antagonists (LTRAs) are a major class of asthma medications, and genetic factors contribute to variable responses to these drugs. We used GWAS to identify novel SNPs associated with the response to the LTRA, montelukast, in asthmatics.

**Methods:**

Using genome-wide genotype and phenotypic data available from American Lung Association - Asthma Clinical Research Center (ALA-ACRC) cohorts, we evaluated 8-week change in FEV_1_ related to montelukast administration in a discovery population of 133 asthmatics. The top 200 SNPs from the discovery GWAS were then tested in 184 additional samples from two independent cohorts.

**Results:**

Twenty-eight SNP associations from the discovery GWAS were replicated. Of these, rs6475448 achieved genome-wide significance (combined P = 1.97 x 10^-09^), and subjects from all four studies who were homozygous for rs6475448 showed increased ΔFEV_1 _from baseline in response to montelukast.

**Conclusions:**

Through GWAS, we identified a novel pharmacogenomic locus related to improved montelukast response in asthmatics.

## Introduction

Two major classes of leukotriene modifiers, including leukotriene antagonists (e.g. montelukast) and lipoxygenase inhibitors (zileuton), are commonly prescribed for management of asthma symptoms. Montelukast [1, 2] targets the cysteinyl leukotriene receptors (CysLTRs) at the cell membrane to block binding of cysteinyl leukotrienes [3], whereas zileuton [4, 5], a 5-lipoxygenase (5-LO) antagonist, exerts its effects upstream of montelukast through inhibition of 5-LO mediated leukotriene biosynthesis from arachidonic acid [6–8]. As with all asthma medications, therapeutic responses to montelukast are highly variable, with some patients responding preferentially to leukotriene modifiers vs. other medications, such as inhaled corticosteroids [9–11]. However, 40–50% of patients do not respond to this class of medication and require additional therapeutic intervention [12]. Mounting evidence suggests that this heterogeneity in treatment response to montelukast is due, in part, to patient genetics [10, 13–15].

To date, multiple genes within the leukotriene pathway, in addition to networks for immune response, have been implicated in differential treatment responses to montelukast, including: *corticotrophin releasing hormone receptor 1* (*CRHR1*) [16, 17], *histone deacetylase 2 (HDAC2) [18], arachidonate 5-lipoxygenase* (*ALOX5*) [10, 11, 13, 14, 16, 19], *arachidonate 5-lipoxygenase-activating protein* (*ALOX5AP)* (20–22), *cysteinyl leukotriene receptor 2* (*CYSLTR2)* [13, 16], *ATP-binding cassette*, *sub-family C (CFTR/MRP)*, *member 1 (ABCC1)* [10, 16], *leukotriene A4 hydrolase (LTA4H) [19–22], leukotriene C4 synthase (LTC4S*) [13, 14, 16, 19, 23], *solute carrier organic anion transporter family*, *member 2B1 (SLCO2B1)* [16, 24], *thromboxane A2 receptor (TBXA2R*) [25–27], *prostaglandin D2 receptor (DP) (PTGDR*) [23], and *interleukin 13 (IL-13)* [28]. However, evidence for genetic associations with montelukast treatment response are available only from candidate gene studies, and additional pharmacogenetic loci for montelukast likely remain undiscovered.

We hypothesized that we could identify novel loci associated with montelukast response using a GWAS approach. We first tested our hypothesis in a discovery GWAS using genotype and phenotype data from two montelukast treatment arms of the Leukotriene Modifier or Corticosteroid or Corticosteroid-Salmeterol (LOCCS) trial [29] and Effectiveness of Low Dose Theophylline as Add On Therapy for the Treatment of Asthma (LODO) trial [1]. We then tested our top SNP associations for replication in two independent cohorts taking montelukast from the Childhood Asthma Research and Education (CARE) Network trials, the Characterizing the Response to a LT Receptor Antagonist and Inhaled Corticosteroid (CLIC) trial [30] and the Pediatric Asthma Controller Trial (PACT) [31].

## Materials and Methods

### Clinical Cohorts and Phenotyping

The discovery cohort included two asthmatic clinical trials with treatment arms evaluating montelukast response, the American Lung Association Asthma Clinical Research Center (ALA-ACRC)-supported trials, the Leukotriene Modifier Or Corticosteroid or Corticosteroid-Salmeterol Trial (LOCCS) and Effectiveness of Low Dose Theophylline as Add On Therapy for the Treatment of Asthma (LODO) [1, 29]. While the LOCCS and LODO clinical trials each analyzed over 400 subjects, for this study, we evaluated a sub-population consisting only of the montelukast treatment arms from these studies that consisted of 133 individuals. For replication, publicly archived, genome-wide SNP data and clinical phenotype information from patients taking montelukast as part of the Childhood Asthma Research and Education (CARE) Network- Characterizing the Response to a LT Receptor Antagonist and an Inhaled Corticosteroid and Pediatric Asthma Controller Trial (CLIC and PACT) (30, 31) (total sample size = 184), were used (dbGaP Study Accession: phs000166.v2.p1 (http://www.ncbi.nlm.nih.gov/projects/gap/cgi-bin/study.cgi?study_id=phs000166.v2.p1)). The data evaluated in this study were obtained from four previously published clinical trials (clinicaltrials.gov identifiers: NCT00156819 (LOCCS); NCT00046644 (LODO); NCT00272506 (PACT); NCT00000622 (CLIC)) [1, 29–31]. Study participants for these trials provided written informed consent, and this consent procedure was approved by the institutional ethics committee/IRB. The Brigham and Women’s Hospital Institutional Review Board approved this study. For all cohorts, subjects were consented for genetic studies and their data was de-identified. Table 1 provides a summary of the populations evaluated in this analysis.

**Table 1:** Demographic information for the clinical cohorts evaluated in this study.

	LOCCS	LODO	CLIC	PACT
N	64	69	126	58
Age, mean yrs. (SD)	35.2 (14.9)	40 (15)	11.7 (3.4)	9.9 (2.3)
Sex- male %	38.9	30.6	40.6	40.2
% European	64.3	68.3	53.7	56.7
% African	8.3	7.3	20.2	13.3
% Asian	27.4	24.4	26.1	30
Mean (SD) change in FEV_1_, mL	11 (32.9)	21.1 (30.5)	1.9 (10)	2.5 (9.1)

Definition of abbreviations: N = number of subjects providing DNA samples evaluated in this study; SD = standard deviation; FEV_1_ = forced expiratory volume in 1 second (mL); LOCCS = Leukotriene Modifier Or Corticosteroid or Corticosteroid-Salmeterol Trial; LODO = Effectiveness of Low Dose Theophylline as Add On Therapy for the Treatment of Asthma; CLIC = Characterizing the Response to a LT Receptor Antagonist and Inhaled Corticosteroid trial; PACT = Pediatric Asthma Controller Trial.

For all populations, the primary outcome phenotype was defined as a change in FEV_1_ following 8 weeks of treatment while on montelukast, minus FEV_1_ at baseline (ΔFEV_1_), adjusted for age, gender, and race.

### Genotyping and Quality Control (QC)

Genome-wide genotyping of the LOCCS and LODO trials was conducted using the Illumina HumanHap550 chip (San Diego, CA). For CLIC and PACT, genotyping was performed as described (30, 31), using the Genomewide Affymetrix SNP 6.0 Array (Santa Clara, CA). The software PLINK v.1.07 [32] was used for QC of genotype data. SNPs with a study-wise missing data proportion above 0.05 were removed from the analysis. SNPs failing to meet Hardy-Weinberg equilibrium (HWE) (P < 0.0001), in addition to SNPs with a minor allele frequency (MAF) < 5% and more than 10% missing genotypes, were also dropped from the analysis. A total of 532,264 SNPs with acceptable quality were genotyped and analyzed in the discovery GWAS for both LOCCS and LODO, and 591,268 SNPs were genotyped and analyzed in both CLIC and PACT.

### Statistical Analysis

For the GWAS, an additive genetic association model was evaluated, adjusting for baseline FEV_1_, age, race (self-reported ancestry) and gender as covariates, using PLINK. Due to small sample sizes, both white and non-white subjects were included. However, the genomic inflation factor values for the subset of montelukast treated patients in these populations was 1, indicating that minimal population stratification was present despite population racial heterogeneity. Due to differences in genotyping platforms used, our analysis focused on the SNPs that were genotyped in all four populations. For replication, the one-sided association P values from 261,076 SNPs that had the same direction of effect in the LOCCS and LODO discovery cohorts were combined, and the top 200 SNPs (as ranked by combined P values) were then carried forward for replication in CLIC and PACT. The one-sided P values of the SNPs that had the same direction of effect (β) in LOCCS-LODO and at least one replication cohort, and that also met nominal significance (P < 0.05) [33, 34] in at least one replication cohort, were combined using a weighted Z-test [35] in ‘R version 3.0.2’ (http://www.r-project.org). SNPs with combined P values below the multiple test correction threshold (P = 0.00025) were considered to be replicated. The threshold for genome-wide significance for associated SNPs was determined using the Bonferroni correction (P = 9.40 x 10^−08^). SNP P values below 10^−05^ were considered suggestive of genome-wide significance.

## Results

**Fig 1:**
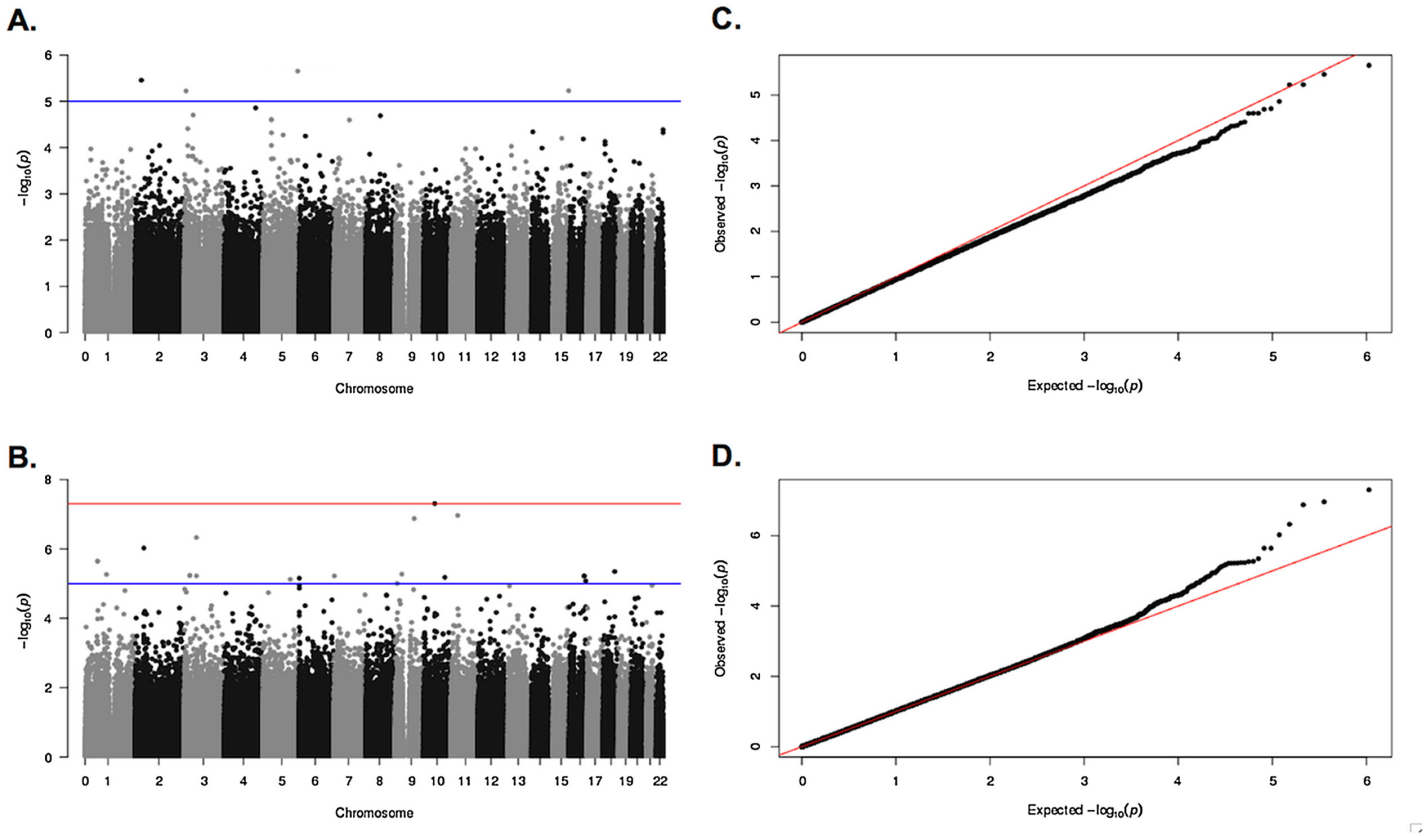
Results of the discovery GWAS. Manhattan plots (**A** and **B**) contain −log P values (y-axis) associated with 8-week change in FEV_1_ after montelukast treatment, for 532,264 genotyped SNPs organized by chromosome (x-axis), for LOCCS (**A**) and LODO (**B**). The threshold for genome-wide significance and suggestive genome-wide significance are indicated as blue and red lines, respectively, in the Manhattan plots. Q-Q plots (**C** and **D**) demonstrate the observed −log P values vs. expected −log P values, for SNPs from LOCCS (**C**) and LODO (**D**) populations. In all plots, individual SNPs are represented as filled circles.

The discovery GWAS was conducted in LOCCS and LODO asthmatic cohorts to evaluate the association of patient genotype with 8-week ΔFEV_1_ following treatment with montelukast (133 patients). Plotted results of the discovery GWAS are shown in Fig 1. Non-white subjects were included, and after adjusting for age, race and gender as covariates, plots of the genomic-control adjusted P values demonstrated no evidence of population stratification. In LOCCS, none of the SNPs exceeded the threshold for genome-wide significance (P = 9.40 x 10^−08^); however, 25 SNPs approached genome-wide significance (P<10^−05^), of which the top-ranked SNP (rs12659144) achieved a P value of 2.2 x 10^−06^, although it did not also replicate in LODO. In LODO, one SNP, rs2247977, achieved genome-wide significance (P = 4.95 x 10^−08^), although it did not also replicate in LOCCS.

For replication of the discovery SNP associations, the P values of the SNPs with the same direction of effect in LOCCS and LODO were combined, and the top-ranked 200 SNPs from LOCCS-LODO were carried forward for evaluation in CLIC and PACT (S1 Table). Four SNPs, s6475448, rs7794356, rs953977 and rs1364805, survived correction for multiple testing (combined P < 0.00025) (Table 2). Three of these SNPs, rs6475448, rs7794356, and rs953977, also approached or achieved genome-wide significance (Table 2).

**Table 2:** Replicated* GWAS SNPs.

					LOCCS		LODO		CLIC		PACT		
SNP	Minor Allele	Chr.	Chr. Location	Gene Symbol	β (mL)	*P* value	β (mL)	*P* value	β (mL)	*P* value	β (mL)	*P* value	Joint *P* Value^‡^
rs6475448	A	9	20487142	*MLLT3*	187	1.22x10^-04^	23.7	3.08x10^-01^	129	4.62 x10^-05^	57.6	3.29 x10^-02^	1.97 x10^-09^
rs7794356	A	7	70376665	*WBSCR17*	215	2.86x10^-04^	47.8	1.39 x10^-01^	110	1.69 x10^-04^	25.3	2.04 x10^-01^	9.15 x10^-07^
rs953977	T	13	39598622		-150	5.57x10^-03^	-116	2.49 x10^-03^	-85.8	4.48 x10^-03^	-41.1	1.25 x10^-01^	5.26 x10^-05^
rs1364805	T	4	107893297		55.1	1.57x10^-01^	154	1.45 x10^-04^	74.9	6.07 x10^-03^	25.1	1.80 x10^-01^	1.69 x10^-04^

Definition of abbreviations: “SNP” = single nucleotide polymorphism; “Chr.” = chromosome (1–22); “Chr. Location.” = chromosomal position of listed SNP; “β” = effect size estimates (ΔFEV_1_, (mL)) for the minor allele.

*Table lists GWA results adjusted for baseline FEV_1_, age, race and gender as covariates (additive genetic model), for the SNPs that met criteria for replication in all cohorts (see Methods) and remained significant after correction for multiple testing. Minor allele frequencies for all SNPs in all cohorts is >5%.

^**‡**^Combined P value for all cohorts.

**Fig 2:**
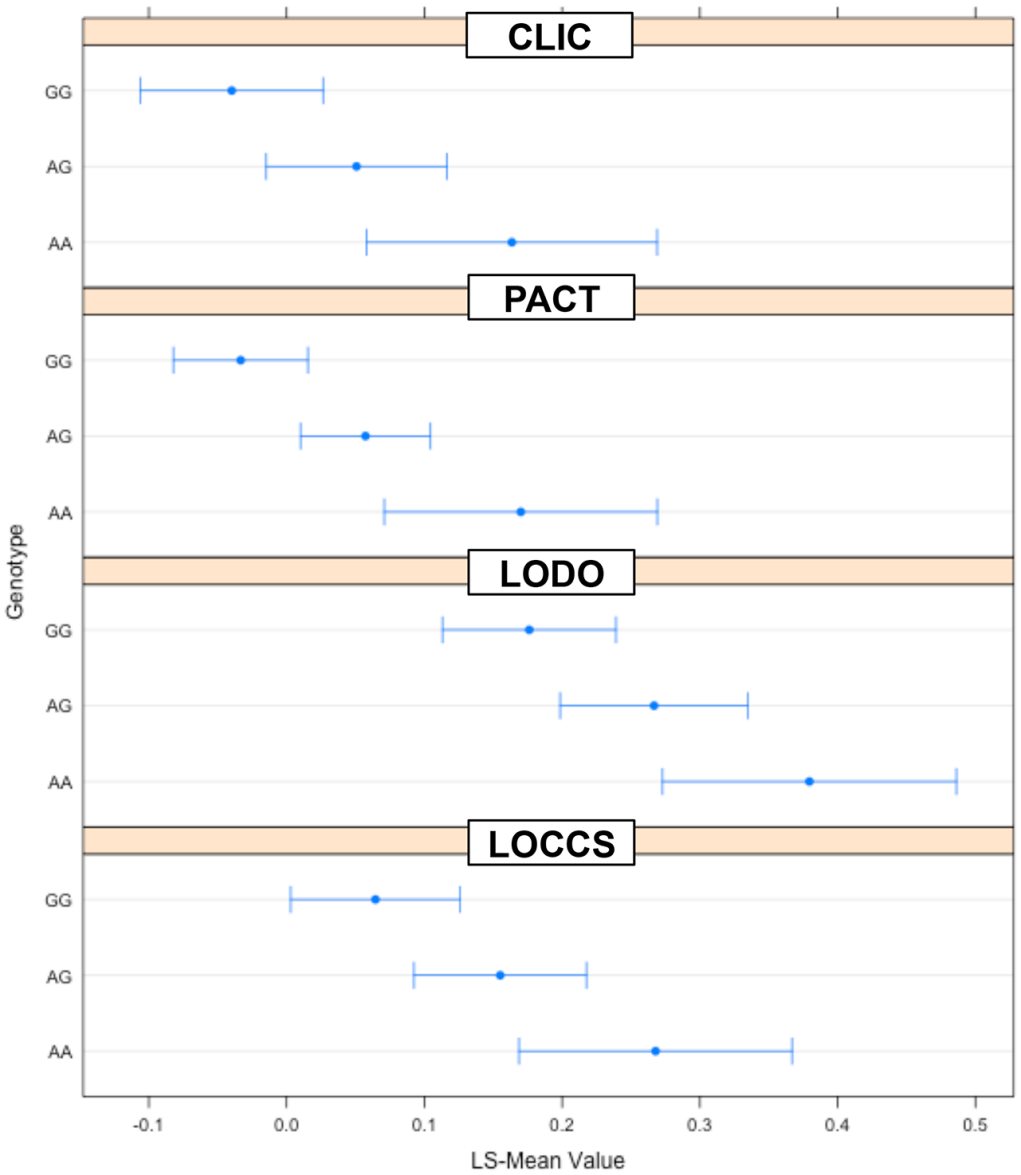
Improvement in lung function related to montelukast treatment, by rs6475448 genotype. The least-squares (LS) means (adjusted for study, race and gender) and 95% confidence intervals for ΔFEV_1_ related to montelukast treatment were generated using R (http://cran.r-project.org/web/packages/lsmeans/lsmeans.pdf), and plotted for each study (panels), by rs6475448 genotypes: homozygous reference (“GG”: LOCCS = 32; LODO = 38; CLIC = 25; PACT = 65), heterozygous (“GA”: LOCCS = 28; LODO = 21; CLIC = 30; PACT = 75) and homozygous variant (“AA”: LOCCS = 9; LODO = 5; CLIC = 5; PACT = 5).

The top-ranked SNP, rs6475448, achieved genome-wide significance (combined P = 1.97 x 10^−09^) (Table 2). Patients from all four studies who were homozygous for rs6475448 showed markedly increased mean ΔFEV_1_ from baseline in response to montelukast (Fig 2). The largest increase between the variant homozygous and reference genotypes was observed for LOCCS, wherein the rs6475448-AA was associated with a LS-mean ΔFEV_1_ of 344 mL vs. -4.66 mL for rs6475448-GG, followed by CLIC (285 mL for rs6475448-AA vs. -31.7 mL for rs6475448-GG), PACT (101 mL for rs6475448-AA vs. -10.6 mL for rs6475448-GG) and LODO (172 mL for rs6475448-AA vs. 192 mL for rs6475448-GG) (Fig 2).

## Discussion

Leukotriene modifier drugs represent a major treatment modality for asthma patients, and the ability of physicians to determine which patients are likely to benefit from these drugs would greatly enhance therapeutic outcomes for asthmatics. We undertook a genome-wide interrogation of 532,264 SNPs to evaluate association of genotype with 8-week ΔFEV_1_ following treatment with montelukast in four asthma clinical trials (LOCCS, LODO, CLIC and PACT). We identified four SNPs that replicated in LOCCS-LODO, CLIC and PACT, of which one variant, rs6475448, achieved genome-wide significance (combined P = 1.97 x 10^−09^) (Table 2). rs6475448 was a novel locus associated with an improvement in response to montelukast in four independent asthmatic populations.

rs6475448 is present within *MLLT3*, which is proposed to regulate cell fates for megakaryocytes and early erythroid cells in humans [36]. Functional and molecular studies have also shown that *MLLT3* acts as a positive regulator of erythroid and megakaryocyte differentiation [36]. Red blood cell precursors including megakaryocytes and erythroid cells are capable of transforming arachidonate and LTA_4_ to bioactive eicosanoids [37, 38]. Megakaryocytes give rise to platelets, which are also activated in asthmatics and contribute to leukotriene production during inflammation [39]. In our study, rs6475448 was associated with a genotype-dependent improved response to montelukast in LOCCS-LODO, CLIC and PACT (Table 2 and Fig 2). While the SNP was intronic, and thus *MLLT3* expression was unlikely to be affected, using the web server SCAN [40], we found that this SNP is also an expression quantitative trait locus (*cis*-eQTL) for *SHROOM3*, a gene that encodes a cytoskeleton protein responsible for cellular shape during morphogenesis [41], and can affect this gene’s expression in the HapMap lymphoblastoid cell lines (LCLs). Therefore, rs6475448, and its eQTL, *SHROOM3*, may potentially represent novel candidate loci for asthma, and/or treatment response to leukotriene modifiers.

Our study has several limitations. First, as is common to many pharmacogenomic GWAS, our sample size is modest; however, our sample size is comparable to recently published GWAS of symptomatic response to corticosteroids in asthma [33–34]. In addition, we were able to replicate four SNPs in multiple independent, montelukast-treated populations, providing supportive evidence of true positive associations. Furthermore, because the cohorts evaluated in this study included non-white subjects, racial heterogeneity may also represent a major limitation of the study; however, we accounted for this by including race, age and gender as covariates in our GWAS models, and saw no evidence of population stratification based on genomic inflation factor and Q-Q plot behavior. A third limitation is that the genotyping platforms used to generate the genome-wide genotype data differed among the four cohorts. To overcome this, we focused our analysis on the SNPs in common between platforms. A fourth limitation is that the ages of our replication and discovery populations differed; while LOCCS and LODO evaluated adults, a pediatric population comprised CLIC and PACT montelukast cohorts. While this supports the generalizability of the reported associations, one reason for failure to replicate additional loci may lie in the innate differences in response between children and adults. For instance, we recently described a pharmacogenetic locus for corticosteroid response [33] that was replicably associated in children, but not in adults. A fifth limitation is that, while we were able to identify a novel montelukast treatment-related gene through GWAS, we did not also find SNPs in reported candidate genes for montelukast response (e.g. *CYSLTR1*) from among the replicated SNP data, which could reflect a limitation of the sensitivity of GWAS, in addition to differences in genotyping platforms used in this study. Finally, additional mechanistic and functional studies will be necessary in order to discern the potential role of *MLLT3* in montelukast response.

## Conclusions

Through a GWAS of differential montelukast response in four asthmatic cohorts, we have identified a genome-wide significant SNP, rs6475448, which is present within *MLLT3*. This SNP may represent a novel mechanism for differential responses to leukotriene modifying agents in asthma.

## Supporting Information

S1 Table Table of top 200 SNPs from LOCCS-LODO tested for replication in CLIC and PACT. (DOCX)
