# Supplementary material for: Genome-Wide Association Study Identifies Novel Pharmacogenomic Loci For Therapeutic Response to Montelukast in Asthma
Source: PLoS One. 2015 Jun 17;10(6):e0129385. doi: 10.1371/journal.pone.0129385 (PMC4470685; doi:10.1371/journal.pone.0129385)
Supplement: S1 Table — (DOCX) [file pone.0129385.s001.docx]

**Supplemental Table 1. Top 200 SNPs From Discovery GWAS Carried Forward for Replication.**

| SNP | Chr. | Chr. Location | Gene Symbol | SNP Location | LOCCS | | LODO | | Combined P Value**^‡^** |
| --- | --- | --- | --- | --- | --- | --- | --- | --- | --- |
|  |  |  |  |  | β (mL) | P value | β (mL) | P value |  |
| rs201260 | 6 | 10102375 | *OFCC1* | intronic | 175.8 | 4.29X10^-04^ | 121.8 | 8.49X10^-04^ | 2.34X10^-06^ |
| rs17035810 | 4 | 158293953 | *GLRB* | intronic | 215.1 | 8.82X10^-03^ | 228.4 | 5.59X10^-05^ | 6.28X10^-06^ |
| rs6084432 | 20 | 3591653 | *GFRA4* | intronic | 200.0 | 2.42X10^-03^ | 199.3 | 4.31X10^-04^ | 7.41X10^-06^ |
| rs12567014 | 1 | 61619670 | *NFIA* | intronic | 116.0 | 8.73X10^-02^ | 285.2 | 1.13X10^-06^ | 1.27X10^-05^ |
| rs829696 | 2 | 30504584 |  |  | 199.5 | 1.06X10^-03^ | 170.2 | 2.31X10^-03^ | 1.46X10^-05^ |
| rs1561629 | 12 | 60686406 | *FAM19A2* | intronic | 349.9 | 7.02X10^-03^ | 331.3 | 2.56X10^-04^ | 1.57X10^-05^ |
| rs6027801 | 20 | 58762495 |  |  | 233.3 | 8.52X10^-03^ | 200.9 | 2.64X10^-04^ | 2.01X10^-05^ |
| rs2433642 | 12 | 22883808 |  |  | 140.8 | 3.60X10^-03^ | 128.0 | 1.11X10^-03^ | 2.55X10^-05^ |
| rs3813383 | 7 | 22200338 | *RAPGEF5* | intronic | 151.1 | 1.44X10^-02^ | 199.7 | 2.48X10^-04^ | 3.58X10^-05^ |
| rs9381279 | 6 | 44107675 |  |  | 148.6 | 1.69X10^-02^ | 209.9 | 2.03X10^-04^ | 3.73X10^-05^ |
| rs10505027 | 8 | 103445553 | *UBR5* | intronic | 157.3 | 8.17X10^-02^ | 266.9 | 1.08X10^-05^ | 4.60X10^-05^ |
| rs7047552 | 9 | 25646011 |  |  | 213.4 | 7.26X10^-03^ | 174.3 | 1.01X10^-03^ | 4.99X10^-05^ |
| rs8086752 | 18 | 41262585 | *SLC14A2* | intronic | 181.7 | 2.52X10^-04^ | 80.2 | 2.71X10^-02^ | 5.67X10^-05^ |
| rs6547580 | 2 | 84980703 |  |  | -235.2 | 5.93X10^-05^ | -68.6 | 6.39X10^-02^ | 5.72X10^-05^ |
| rs16941252 | 15 | 86439010 | *NTRK3* | intronic | 152.2 | 3.33X10^-02^ | 235.8 | 1.58X10^-04^ | 7.41X10^-05^ |
| rs17172171 | 7 | 43197029 | *HECW1* | intronic | 242.5 | 1.29X10^-02^ | 190.4 | 7.85X10^-04^ | 7.73X10^-05^ |
| rs7500931 | 16 | 49837325 |  |  | 178.4 | 3.75X10^-04^ | 73.2 | 2.69X10^-02^ | 7.75X10^-05^ |
| rs953977 | 13 | 39598622 |  |  | -149.7 | 5.57X10^-03^ | -116.2 | 2.49X10^-03^ | 8.15X10^-05^ |
| rs12504273 | 4 | 152768562 | *FAM160A1* | intronic | 215.3 | 2.01X10^-02^ | 283.1 | 4.35X10^-04^ | 8.19X10^-05^ |
| rs2958089 | 3 | 112483706 |  |  | 151.0 | 4.42X10^-02^ | 348.8 | 1.06X10^-04^ | 8.20X10^-05^ |
| rs1718125 | 12 | 120077402 | *P2RX7* | intronic | 225.4 | 1.74X10^-03^ | 135.0 | 8.61X10^-03^ | 8.41X10^-05^ |
| rs7020934 | 9 | 34251117 | *KIF24* | intronic | -192.4 | 5.44X10^-04^ | -81.4 | 2.44X10^-02^ | 9.40X10^-05^ |
| rs4984338 | 15 | 90446375 | *SLCO3A1* | intronic | -294.9 | 1.26X10^-04^ | -80.2 | 6.26X10^-02^ | 9.69X10^-05^ |
| rs4483828 | 15 | 97892693 |  |  | 222.1 | 2.95X10^-06^ | 20.5 | 2.95X10^-01^ | 1.13X10^-04^ |
| rs855399 | 6 | 10155458 | *OFCC1* | intronic | -156.3 | 8.79X10^-03^ | -128.4 | 2.11X10^-03^ | 1.14X10^-04^ |
| rs10500202 | 7 | 151212341 |  |  | 142.7 | 6.66X10^-02^ | 455.0 | 7.27X10^-05^ | 1.15X10^-04^ |
| rs2892978 | 7 | 20275903 |  |  | -101.5 | 3.70X10^-02^ | -143.8 | 2.45X10^-04^ | 1.16X10^-04^ |
| rs956578 | 9 | 108945434 |  |  | 190.0 | 1.16X10^-02^ | 168.8 | 1.54X10^-03^ | 1.18X10^-04^ |
| rs227811 | 6 | 44779065 |  |  | -259.1 | 5.05X10^-03^ | -173.9 | 4.56X10^-03^ | 1.27X10^-04^ |
| rs17130969 | 1 | 90328738 |  |  | 86.3 | 1.22X10^-01^ | 255.8 | 1.99X10^-05^ | 1.31X10^-04^ |
| rs10733789 | 10 | 64618690 | *JMJD1C* | intronic | 180.4 | 4.00X10^-04^ | 82.4 | 4.18X10^-02^ | 1.39X10^-04^ |
| rs2454909 | 16 | 31318449 | *ITGAD* | intronic | 200.9 | 1.02X10^-02^ | 147.6 | 2.26X10^-03^ | 1.41X10^-04^ |
| rs10114302 | 9 | 137858802 | *CAMSAP1* | intronic | -261.0 | 1.62X10^-03^ | -131.7 | 1.68X10^-02^ | 1.57X10^-04^ |
| rs2692341 | 17 | 10783644 |  |  | 183.7 | 6.62X10^-03^ | 185.9 | 4.35X10^-03^ | 1.59X10^-04^ |
| rs1316453 | 17 | 74922454 | *RBFOX3* | intronic | 105.9 | 2.01X10^-02^ | 141.3 | 1.10X10^-03^ | 1.68X10^-04^ |
| rs1420987 | 16 | 49940531 |  |  | -139.1 | 3.79X10^-03^ | -116.9 | 9.05X10^-03^ | 1.82X10^-04^ |
| rs1653583 | 12 | 120083035 | *P2RX7* | intronic | 227.8 | 8.58X10^-03^ | 177.6 | 3.85X10^-03^ | 1.85X10^-04^ |
| rs7257553 | 19 | 44738703 |  |  | 136.6 | 6.66X10^-03^ | 132.4 | 5.28X10^-03^ | 1.90X10^-04^ |
| rs9938025 | 16 | 70579542 | *PKD1L3* | intronic | 165.0 | 6.31X10^-04^ | 70.5 | 4.15X10^-02^ | 1.98X10^-04^ |
| rs1473070 | 4 | 184429031 | *WWC2* | intronic | 156.3 | 1.12X10^-03^ | 77.8 | 2.85X10^-02^ | 2.04X10^-04^ |
| rs149340 | 13 | 49959045 | *DLEU1* | intronic | 217.3 | 1.41X10^-04^ | 59.8 | 1.03X10^-01^ | 2.14X10^-04^ |
| rs10892640 | 11 | 120294718 | *GRIK4* | intronic | 178.3 | 2.82X10^-04^ | 58.7 | 7.85X10^-02^ | 2.41X10^-04^ |
| rs6103533 | 20 | 42017812 | *TOX2* | intronic | -190.8 | 6.81X10^-04^ | -70.4 | 4.72X10^-02^ | 2.45X10^-04^ |
| rs2244964 | 5 | 10316725 | *CCT5* | intronic | -103.5 | 6.11X10^-02^ | -148.1 | 2.74X10^-04^ | 2.48X10^-04^ |
| rs11875257 | 18 | 6946393 | *LAMA1* | intronic | 76.9 | 8.12X10^-02^ | 154.3 | 1.58X10^-04^ | 2.56X10^-04^ |
| rs10768011 | 11 | 33594964 | *KIAA1549L* | intronic | 129.2 | 1.04X10^-02^ | 106.4 | 4.61X10^-03^ | 2.65X10^-04^ |
| rs4787348 | 16 | 26002100 | *HS3ST4* | intronic | -249.4 | 2.78X10^-03^ | -147.2 | 1.76X10^-02^ | 2.65X10^-04^ |
| rs13297172 | 9 | 7468453 |  |  | 118.7 | 7.31X10^-02^ | 257.0 | 2.30X10^-04^ | 2.83X10^-04^ |
| rs693250 | 18 | 58792327 | *PHLPP1* | intronic | 49.9 | 3.28X10^-01^ | 358.4 | 2.25X10^-06^ | 2.85X10^-04^ |
| rs4314501 | 6 | 57966759 |  |  | -157.0 | 2.62X10^-03^ | -92.4 | 2.02X10^-02^ | 2.90X10^-04^ |
| rs12505156 | 4 | 119334814 | *NDST3* | intronic | 110.4 | 7.48X10^-02^ | 180.7 | 2.29X10^-04^ | 2.91X10^-04^ |
| rs4075464 | 16 | 81706187 | *CDH13* | intronic | -174.4 | 7.46X10^-04^ | -71.9 | 5.26X10^-02^ | 3.01X10^-04^ |
| rs10267957 | 7 | 34980824 | *DPY19L1* | intronic | 106.7 | 3.24X10^-02^ | 187.0 | 1.14X10^-03^ | 3.03X10^-04^ |
| rs4478536 | 8 | 129433393 |  |  | 199.6 | 5.24X10^-03^ | 133.6 | 1.17X10^-02^ | 3.15X10^-04^ |
| rs409228 | 3 | 41040417 |  |  | 74.7 | 8.50X10^-02^ | 140.2 | 1.95X10^-04^ | 3.16X10^-04^ |
| rs3799936 | 6 | 62400202 | *KHDRBS2* | intronic | -162.4 | 2.78X10^-03^ | -100.9 | 2.09X10^-02^ | 3.16X10^-04^ |
| rs2076530 | 6 | 32471794 | *BTNL2* | missense | -194.9 | 1.25X10^-04^ | -41.7 | 1.43X10^-01^ | 3.24X10^-04^ |
| rs9315923 | 13 | 41968894 |  |  | -121.0 | 1.50X10^-02^ | -118.0 | 3.78X10^-03^ | 3.28X10^-04^ |
| rs201247 | 6 | 10122547 | *OFCC1* | intronic | -130.3 | 1.10X10^-02^ | -115.4 | 5.53X10^-03^ | 3.30X10^-04^ |
| rs13165359 | 5 | 40271560 |  |  | 218.1 | 8.16X10^-03^ | 163.8 | 7.83X10^-03^ | 3.32X10^-04^ |
| rs7082582 | 10 | 113981387 |  |  | 146.9 | 7.46X10^-03^ | 98.1 | 8.90X10^-03^ | 3.41X10^-04^ |
| rs12148280 | 15 | 55563118 | *CGNL1* | intronic | 235.9 | 7.63X10^-03^ | 137.3 | 9.22X10^-03^ | 3.60X10^-04^ |
| rs12965086 | 18 | 5727924 |  |  | 275.7 | 5.42X10^-03^ | 162.8 | 1.30X10^-02^ | 3.61X10^-04^ |
| rs6054464 | 20 | 6620360 |  |  | 153.1 | 2.33X10^-03^ | 73.1 | 2.75X10^-02^ | 3.63X10^-04^ |
| rs2827784 | 21 | 23261439 |  |  | 181.0 | 6.07X10^-04^ | 66.7 | 6.97X10^-02^ | 3.66X10^-04^ |
| rs4121759 | 11 | 100596067 |  |  | -295.0 | 7.75X10^-04^ | -119.0 | 6.03X10^-02^ | 3.67X10^-04^ |
| rs13242837 | 7 | 19166849 |  |  | 153.9 | 3.12X10^-03^ | 97.8 | 2.23X10^-02^ | 3.75X10^-04^ |
| rs9787186 | 1 | 90876473 |  |  | 159.1 | 6.99X10^-03^ | 126.0 | 1.08X10^-02^ | 3.85X10^-04^ |
| rs2090380 | 4 | 154205775 |  |  | -125.7 | 6.92X10^-02^ | -164.8 | 4.12X10^-04^ | 3.88X10^-04^ |
| rs1020407 | 2 | 209912715 |  |  | 98.5 | 3.23X10^-02^ | 157.5 | 1.64X10^-03^ | 3.97X10^-04^ |
| rs1538515 | 9 | 12257378 |  |  | 123.7 | 3.60X10^-02^ | 121.8 | 1.39X10^-03^ | 4.00X10^-04^ |
| rs17011040 | 2 | 124660578 | *CNTNAP5* | intronic | -205.4 | 5.64X10^-04^ | -64.5 | 7.79X10^-02^ | 4.00X10^-04^ |
| rs1464615 | 3 | 47628706 | *SMARCC1* | intronic | 140.2 | 7.27X10^-02^ | 274.2 | 3.90X10^-04^ | 4.00X10^-04^ |
| rs11243356 | 9 | 133213012 |  |  | 163.4 | 3.75X10^-02^ | 161.5 | 1.31X10^-03^ | 4.00X10^-04^ |
| rs17412577 | 1 | 100882019 | *RP11-84O12.4* | intronic | 172.1 | 4.49X10^-02^ | 276.8 | 9.75X10^-04^ | 4.04X10^-04^ |
| rs4815931 | 20 | 6626538 |  |  | 139.8 | 5.70X10^-03^ | 89.6 | 1.40X10^-02^ | 4.05X10^-04^ |
| rs10519404 | 4 | 138094899 |  |  | 153.9 | 5.71X10^-03^ | 104.7 | 1.43X10^-02^ | 4.13X10^-04^ |
| rs13204006 | 6 | 22054591 | *CASC15* | intronic | 207.8 | 2.57X10^-03^ | 88.4 | 2.91X10^-02^ | 4.19X10^-04^ |
| rs8075188 | 17 | 66898753 |  |  | -207.2 | 3.65X10^-04^ | -52.1 | 1.03X10^-01^ | 4.21X10^-04^ |
| rs3753539 | 1 | 169982808 |  |  | 84.8 | 7.55X10^-02^ | 148.8 | 3.91X10^-04^ | 4.23X10^-04^ |
| rs11124763 | 2 | 40891407 |  |  | -100.1 | 2.64X10^-02^ | -111.0 | 2.44X10^-03^ | 4.28X10^-04^ |
| rs2697684 | 4 | 17045056 |  |  | 181.1 | 5.29X10^-04^ | 75.8 | 8.68X10^-02^ | 4.40X10^-04^ |
| rs9555776 | 13 | 110608828 | *ARHGEF7* | intronic | 111.8 | 1.14X10^-01^ | 258.6 | 1.76X10^-04^ | 4.56X10^-04^ |
| rs723488 | 11 | 123352074 |  |  | 203.3 | 1.66X10^-03^ | 77.1 | 4.41X10^-02^ | 4.63X10^-04^ |
| rs9975358 | 21 | 33501442 |  |  | 186.4 | 6.01X10^-03^ | 116.4 | 1.63X10^-02^ | 4.91X10^-04^ |
| rs7552560 | 1 | 22419877 |  |  | 166.2 | 1.16X10^-03^ | 67.8 | 5.98X10^-02^ | 5.00X10^-04^ |
| rs2043516 | 15 | 86275791 | *NTRK3* | intronic | 113.2 | 2.25X10^-02^ | 145.9 | 3.76X10^-03^ | 5.06X10^-04^ |
| rs784718 | 3 | 131169708 |  |  | 188.0 | 3.20X10^-02^ | 168.0 | 2.27X10^-03^ | 5.06X10^-04^ |
| rs9396778 | 6 | 10084201 | *OFCC1* | intronic | 92.1 | 4.94X10^-02^ | 135.0 | 1.13X10^-03^ | 5.08X10^-04^ |
| rs10223785 | 6 | 1933384 | *GMDS* | intronic | -234.0 | 1.96X10^-02^ | -230.7 | 4.62X10^-03^ | 5.14X10^-04^ |
| rs967597 | 13 | 51389194 | *ATP7B* | intronic | 89.9 | 4.14X10^-02^ | 133.6 | 1.59X10^-03^ | 5.24X10^-04^ |
| rs3744311 | 17 | 67919451 | *LINC00673* | intronic | 76.0 | 8.52X10^-02^ | 128.5 | 4.18X10^-04^ | 5.24X10^-04^ |
| rs4665609 | 2 | 23628257 | *KLHL29* | intronic | -143.9 | 2.31X10^-03^ | -78.6 | 3.94X10^-02^ | 5.34X10^-04^ |
| rs6483253 | 11 | 92946330 |  |  | 294.1 | 1.58X10^-03^ | 138.3 | 5.19X10^-02^ | 5.37X10^-04^ |
| rs16945435 | 12 | 114277647 |  |  | 252.5 | 1.29X10^-03^ | 101.8 | 5.98X10^-02^ | 5.41X10^-04^ |
| rs7794356 | 7 | 70376665 | *WBSCR17* | intronic | 214.7 | 2.86X10^-04^ | 47.8 | 1.39X10^-01^ | 5.48X10^-04^ |
| rs10833416 | 11 | 20942913 | *NELL1* | intronic | 137.6 | 6.29X10^-03^ | 99.1 | 1.76X10^-02^ | 5.53X10^-04^ |
| rs3778995 | 7 | 2142397 | *MAD1L1* | intronic | 167.2 | 6.58X10^-03^ | 135.1 | 1.70X10^-02^ | 5.58X10^-04^ |
| rs8060190 | 16 | 82235804 | *CDH13* | intronic | 27.2 | 3.78X10^-01^ | 268.7 | 4.23X10^-06^ | 5.62X10^-04^ |
| rs12432376 | 14 | 25012669 |  |  | 146.0 | 8.64X10^-03^ | 110.8 | 1.33X10^-02^ | 5.70X10^-04^ |
| rs6958192 | 7 | 49254669 |  |  | -102.2 | 4.97X10^-02^ | -117.1 | 1.33X10^-03^ | 5.74X10^-04^ |
| rs3091249 | 20 | 41789167 | *GTSF1L* | upstream | 101.6 | 7.16X10^-02^ | 213.3 | 7.00X10^-04^ | 5.85X10^-04^ |
| rs915286 | 13 | 39593992 |  |  | -110.8 | 1.39X10^-02^ | -97.1 | 8.36X10^-03^ | 5.96X10^-04^ |
| rs6838273 | 4 | 141167734 | *MAML3* | intronic | -159.2 | 3.30X10^-02^ | -158.3 | 2.71X10^-03^ | 6.01X10^-04^ |
| rs7105756 | 11 | 20939986 | *NELL1* | intronic | 134.0 | 8.56X10^-03^ | 94.9 | 1.42X10^-02^ | 6.03X10^-04^ |
| rs6912306 | 6 | 108101021 |  |  | 109.2 | 7.48X10^-02^ | 184.5 | 6.73X10^-04^ | 6.04X10^-04^ |
| rs6989782 | 8 | 9647948 | *TNKS* | intronic | 112.9 | 2.49X10^-02^ | 123.9 | 4.16X10^-03^ | 6.13X10^-04^ |
| rs7080649 | 10 | 4721081 |  |  | 57.4 | 1.50X10^-01^ | 144.3 | 1.41X10^-04^ | 6.19X10^-04^ |
| rs9890937 | 17 | 4607257 |  |  | -170.1 | 2.08X10^-04^ | -43.0 | 1.74X10^-01^ | 6.26X10^-04^ |
| rs13423991 | 2 | 29312813 | *ALK* | intronic | 181.1 | 1.23X10^-02^ | 133.9 | 1.04X10^-02^ | 6.40X10^-04^ |
| rs716890 | 4 | 43885151 | *KCTD8* | intronic | 122.4 | 3.85X10^-02^ | 162.5 | 2.34X10^-03^ | 6.41X10^-04^ |
| rs6972154 | 7 | 30850798 | *FAM188B* | intronic | -137.8 | 4.27X10^-02^ | -177.3 | 2.04X10^-03^ | 6.54X10^-04^ |
| rs9815806 | 3 | 27990474 |  |  | 164.5 | 9.48X10^-03^ | 134.9 | 1.44X10^-02^ | 6.69X10^-04^ |
| rs1364805 | 4 | 107893297 |  |  | 55.1 | 1.57X10^-01^ | 153.9 | 1.45X10^-04^ | 6.82X10^-04^ |
| rs6665581 | 1 | 169987769 |  |  | 61.2 | 1.28X10^-01^ | 148.6 | 2.49X10^-04^ | 6.83X10^-04^ |
| rs4784274 | 16 | 50011624 |  |  | -90.4 | 4.46X10^-02^ | -103.0 | 2.02X10^-03^ | 6.85X10^-04^ |
| rs7448746 | 5 | 92602250 |  |  | -119.4 | 1.86X10^-02^ | -96.2 | 6.95X10^-03^ | 6.87X10^-04^ |
| rs10960478 | 9 | 12089364 |  |  | 44.2 | 2.55X10^-01^ | 184.8 | 3.43X10^-05^ | 7.11X10^-04^ |
| rs1525636 | 7 | 122996065 | *NDUFA5* | intronic | -98.5 | 2.30X10^-02^ | -108.7 | 5.55X10^-03^ | 7.12X10^-04^ |
| rs199635 | 6 | 72282724 |  |  | 116.1 | 3.74X10^-02^ | 164.4 | 2.80X10^-03^ | 7.12X10^-04^ |
| rs7988392 | 13 | 21680909 | *LINC00540* | upstream | 123.3 | 9.88X10^-03^ | 112.1 | 1.48X10^-02^ | 7.13X10^-04^ |
| rs991183 | 4 | 124393085 | *SPATA5* | intronic | 161.6 | 1.55X10^-02^ | 134.8 | 9.09X10^-03^ | 7.19X10^-04^ |
| rs2892380 | 10 | 7125663 |  |  | 66.3 | 2.32X10^-01^ | 242.0 | 4.91X10^-05^ | 7.19X10^-04^ |
| rs2063492 | 4 | 184468459 | *WWC2* | intronic | 119.9 | 1.15X10^-02^ | 98.1 | 1.28X10^-02^ | 7.24X10^-04^ |
| rs7175947 | 15 | 33168779 |  |  | 102.6 | 8.85X10^-02^ | 145.7 | 6.38X10^-04^ | 7.35X10^-04^ |
| rs6044417 | 20 | 16765706 |  |  | 158.9 | 2.32X10^-03^ | 84.0 | 5.23X10^-02^ | 7.37X10^-04^ |
| rs17521834 | 4 | 215449 | *ZNF876P* | intronic | 152.9 | 9.14X10^-02^ | 223.5 | 6.08X10^-04^ | 7.44X10^-04^ |
| rs1999437 | 9 | 79936404 |  |  | -96.2 | 2.54X10^-02^ | -92.5 | 5.18X10^-03^ | 7.48X10^-04^ |
| rs4943464 | 13 | 36736294 |  |  | -226.1 | 8.32X10^-03^ | -138.6 | 1.87X10^-02^ | 7.61X10^-04^ |
| rs1838733 | 5 | 58569149 | *PDE4D* | intronic | -117.7 | 1.72X10^-02^ | -94.1 | 8.63X10^-03^ | 7.63X10^-04^ |
| rs2204729 | 2 | 51657546 | *AC007682.1* | intronic | -139.1 | 3.74X10^-03^ | -81.7 | 3.83X10^-02^ | 7.74X10^-04^ |
| rs1146815 | 13 | 67702749 |  |  | -46.0 | 1.85X10^-01^ | -136.6 | 1.14X10^-04^ | 7.77X10^-04^ |
| rs17152064 | 10 | 126257749 | *LHPP* | intronic | 130.0 | 1.14X10^-01^ | 185.2 | 4.15X10^-04^ | 7.93X10^-04^ |
| rs6461599 | 7 | 21766984 | *DNAH11* | intronic | -207.4 | 4.54X10^-03^ | -87.4 | 3.39X10^-02^ | 8.02X10^-04^ |
| rs276420 | 13 | 39470070 |  |  | 87.3 | 5.83X10^-02^ | 110.3 | 1.61X10^-03^ | 8.04X10^-04^ |
| rs10771512 | 12 | 29331503 | *FAR2* | intronic | 127.0 | 9.19X10^-03^ | 88.5 | 1.88X10^-02^ | 8.35X10^-04^ |
| rs10935257 | 3 | 138819439 |  |  | 141.3 | 3.75X10^-03^ | 75.7 | 4.11X10^-02^ | 8.36X10^-04^ |
| rs286969 | 5 | 153301961 |  |  | 84.4 | 7.69X10^-02^ | 131.2 | 1.05X10^-03^ | 8.51X10^-04^ |
| rs3844038 | 18 | 41267039 | *SLC14A2* | intronic | 153.4 | 1.79X10^-03^ | 59.5 | 7.06X10^-02^ | 8.56X10^-04^ |
| rs13434426 | 4 | 170184677 |  |  | -195.8 | 1.30X10^-02^ | -112.7 | 1.36X10^-02^ | 8.60X10^-04^ |
| rs305009 | 15 | 67932812 |  |  | -236.3 | 7.31X10^-04^ | -58.8 | 1.20X10^-01^ | 8.63X10^-04^ |
| rs1454398 | 2 | 52753920 |  |  | 131.8 | 2.46X10^-02^ | 98.2 | 6.54X10^-03^ | 8.73X10^-04^ |
| rs10092705 | 8 | 9811668 |  |  | 202.4 | 7.13X10^-03^ | 96.3 | 2.52X10^-02^ | 8.81X10^-04^ |
| rs12905620 | 15 | 22481471 |  |  | -108.7 | 4.99X10^-02^ | -139.5 | 2.43X10^-03^ | 8.99X10^-04^ |
| rs4576132 | 5 | 158916817 |  |  | -99.4 | 2.43X10^-02^ | -101.6 | 6.99X10^-03^ | 9.12X10^-04^ |
| rs6537519 | 10 | 50149830 |  |  | -185.9 | 1.50X10^-02^ | -120.8 | 1.25X10^-02^ | 9.15X10^-04^ |
| rs1640881 | 16 | 6331875 | *RBFOX1* | intronic | -131.5 | 1.62X10^-02^ | -96.7 | 1.14X10^-02^ | 9.18X10^-04^ |
| rs1905760 | 9 | 7702066 |  |  | 129.4 | 8.50X10^-03^ | 78.9 | 2.26X10^-02^ | 9.27X10^-04^ |
| rs3813086 | 18 | 46442790 | *MAPK4* | intronic | -76.4 | 7.53X10^-02^ | -123.8 | 1.25X10^-03^ | 9.33X10^-04^ |
| rs17641650 | 19 | 44770271 |  |  | 130.0 | 1.35X10^-02^ | 115.0 | 1.44X10^-02^ | 9.43X10^-04^ |
| rs2216782 | 2 | 207884477 |  |  | -153.3 | 7.40X10^-03^ | -80.9 | 2.61X10^-02^ | 9.43X10^-04^ |
| rs1146827 | 13 | 67717268 |  |  | -41.8 | 2.06X10^-01^ | -136.6 | 1.14X10^-04^ | 9.43X10^-04^ |
| rs1077758 | 17 | 73120267 |  |  | 161.5 | 3.58X10^-03^ | 78.0 | 4.77X10^-02^ | 9.50X10^-04^ |
| rs17011381 | 2 | 124770724 | *CNTNAP5* | intronic | -249.9 | 1.37X10^-04^ | -38.3 | 2.61X10^-01^ | 9.60X10^-04^ |
| rs8025430 | 15 | 51161318 |  |  | -163.3 | 3.19X10^-04^ | -35.0 | 1.90X10^-01^ | 9.69X10^-04^ |
| rs7153719 | 14 | 82326527 |  |  | -92.1 | 1.91X10^-02^ | -91.6 | 1.01X10^-02^ | 9.76X10^-04^ |
| rs1342806 | 1 | 191011435 |  |  | 128.8 | 9.22X10^-03^ | 88.9 | 2.21X10^-02^ | 9.78X10^-04^ |
| rs2846662 | 18 | 465796 | *COLEC12* | intronic | -81.6 | 7.15X10^-02^ | -122.3 | 1.50X10^-03^ | 9.95X10^-04^ |
| rs6535574 | 4 | 149148251 | *ARHGAP10* | intronic | -231.1 | 1.71X10^-04^ | -32.3 | 2.48X10^-01^ | 1.01X10^-03^ |
| rs4833561 | 4 | 119266722 | *NDST3* | intronic | 63.7 | 1.94X10^-01^ | 192.7 | 1.57X10^-04^ | 1.02X10^-03^ |
| rs9458345 | 6 | 162063314 | *PARK2* | intronic | -56.8 | 1.81X10^-01^ | -177.5 | 1.90X10^-04^ | 1.02X10^-03^ |
| rs1472931 | 2 | 234997470 |  |  | 141.7 | 1.30X10^-02^ | 118.4 | 1.63X10^-02^ | 1.02X10^-03^ |
| rs4722957 | 7 | 29908509 | *WIPF3* | intronic | -63.8 | 1.16X10^-01^ | -139.1 | 5.89X10^-04^ | 1.02X10^-03^ |
| rs7757215 | 6 | 28320468 | *ZKSCAN4* | 3'utr | -222.4 | 8.84X10^-03^ | -121.5 | 2.41X10^-02^ | 1.02X10^-03^ |
| rs9314794 | 9 | 73927257 | *GDA* | intronic | -150.9 | 6.58X10^-03^ | -86.4 | 3.15X10^-02^ | 1.03X10^-03^ |
| rs6986543 | 8 | 128465498 | *LOC101930033* | intronic | -116.3 | 1.44X10^-02^ | -83.0 | 1.49X10^-02^ | 1.04X10^-03^ |
| rs9374658 | 6 | 117848188 | *ROS1* | intronic | -111.8 | 1.81X10^-02^ | -90.9 | 1.18X10^-02^ | 1.05X10^-03^ |
| rs13045227 | 20 | 59559552 | *CDH4* | intronic | 251.0 | 3.89X10^-02^ | 203.9 | 4.37X10^-03^ | 1.05X10^-03^ |
| rs10484565 | 6 | 32903010 | *TAP2* | 3'utr | -232.0 | 1.12X10^-02^ | -189.5 | 1.98X10^-02^ | 1.05X10^-03^ |
| rs17206578 | 11 | 84211479 | *DLG2* | intronic | -205.4 | 1.31X10^-02^ | -230.8 | 1.72X10^-02^ | 1.07X10^-03^ |
| rs639839 | 10 | 84353307 | *NRG3* | intronic | -189.9 | 5.88X10^-03^ | -118.1 | 3.62X10^-02^ | 1.07X10^-03^ |
| rs7749507 | 6 | 134471916 |  |  | 152.2 | 2.36X10^-03^ | 73.3 | 7.12X10^-02^ | 1.07X10^-03^ |
| rs1014137 | 7 | 31590271 | *CCDC129* | intronic | -165.6 | 4.60X10^-04^ | -37.4 | 1.74X10^-01^ | 1.08X10^-03^ |
| rs7536709 | 1 | 232558682 |  |  | -102.7 | 5.90X10^-02^ | -120.2 | 2.38X10^-03^ | 1.09X10^-03^ |
| rs2072020 | 6 | 112567341 | *LAMA4* | intronic | 102.8 | 7.36X10^-02^ | 137.6 | 1.64X10^-03^ | 1.10X10^-03^ |
| rs2015808 | 15 | 90452315 | *SLCO3A1* | intronic | 131.1 | 9.44X10^-03^ | 86.2 | 2.44X10^-02^ | 1.10X10^-03^ |
| rs7592297 | 2 | 206481747 |  |  | 91.4 | 4.09X10^-02^ | 101.3 | 4.36X10^-03^ | 1.11X10^-03^ |
| rs12550796 | 8 | 138627496 |  |  | 124.7 | 6.57X10^-02^ | 176.2 | 2.03X10^-03^ | 1.11X10^-03^ |
| rs12101550 | 15 | 90658346 |  |  | -108.7 | 1.66X10^-02^ | -86.3 | 1.39X10^-02^ | 1.12X10^-03^ |
| rs284780 | 4 | 100523446 | *LOC102723576* | downstream | 223.4 | 1.67X10^-02^ | 220.3 | 1.38X10^-02^ | 1.12X10^-03^ |
| rs2025758 | 10 | 8881675 |  |  | 96.6 | 2.28X10^-02^ | 90.5 | 9.68X10^-03^ | 1.12X10^-03^ |
| rs2303792 | 16 | 69088867 | *COG4* | intronic | 100.3 | 2.37X10^-02^ | 115.4 | 9.28X10^-03^ | 1.12X10^-03^ |
| rs598444 | 18 | 2163591 |  |  | 67.3 | 9.94X10^-02^ | 130.4 | 9.48X10^-04^ | 1.13X10^-03^ |
| rs1978759 | 10 | 131392438 | *MGMT* | intronic | 155.4 | 2.09X10^-02^ | 104.0 | 1.08X10^-02^ | 1.13X10^-03^ |
| rs9310356 | 3 | 76298335 | *ROBO2* | intronic | -122.5 | 3.40X10^-02^ | -134.0 | 5.82X10^-03^ | 1.13X10^-03^ |
| rs11044770 | 12 | 19732926 |  |  | -174.7 | 1.06X10^-02^ | -145.8 | 2.24X10^-02^ | 1.13X10^-03^ |
| rs1997021 | 18 | 25930660 |  |  | 128.2 | 2.30X10^-02^ | 104.1 | 9.79X10^-03^ | 1.14X10^-03^ |
| rs11563992 | 7 | 27347461 |  |  | 120.1 | 1.12X10^-01^ | 232.1 | 7.56X10^-04^ | 1.15X10^-03^ |
| rs4907110 | 1 | 85143853 |  |  | 87.5 | 4.58X10^-02^ | 100.3 | 3.96X10^-03^ | 1.17X10^-03^ |
| rs12513198 | 4 | 166501454 |  |  | 179.0 | 9.71X10^-04^ | 64.9 | 1.29X10^-01^ | 1.17X10^-03^ |
| rs6102757 | 20 | 40339912 | *PTPRT* | intronic | 148.4 | 8.42X10^-03^ | 91.2 | 2.90X10^-02^ | 1.18X10^-03^ |
| rs11642627 | 16 | 6340689 | *RBFOX1* | intronic | -200.0 | 1.50X10^-02^ | -120.1 | 1.65X10^-02^ | 1.18X10^-03^ |
| rs1479457 | 12 | 23689385 | *SOX5* | intronic | -175.3 | 2.46X10^-03^ | -74.2 | 7.51X10^-02^ | 1.18X10^-03^ |
| rs3857946 | 8 | 125675435 | *MTSS1* | intronic | 160.3 | 5.20X10^-02^ | 170.2 | 3.30X10^-03^ | 1.19X10^-03^ |
| rs1921045 | 12 | 80239063 | *PPFIA2* | intronic | 107.3 | 1.78X10^-02^ | 106.3 | 1.38X10^-02^ | 1.19X10^-03^ |
| rs4610277 | 4 | 5720825 | *EVC2* | intronic | -123.9 | 2.40X10^-02^ | -105.8 | 9.82X10^-03^ | 1.20X10^-03^ |
| rs11106513 | 12 | 91251187 |  |  | -153.7 | 1.71X10^-02^ | -113.8 | 1.47X10^-02^ | 1.20X10^-03^ |
| rs5995172 | 22 | 34580704 | *RBFOX2* | intronic | -68.0 | 1.81X10^-01^ | -181.1 | 2.50X10^-04^ | 1.20X10^-03^ |
| rs6475448 | 9 | 20487142 | *MLLT3* | intronic | 186.7 | 1.22X10^-04^ | 23.7 | 3.08X10^-01^ | 1.22X10^-03^ |
| rs4834827 | 4 | 121077628 | *RP11-700N1.1* | intronic | -136.7 | 4.84X10^-03^ | -60.4 | 4.81X10^-02^ | 1.23X10^-03^ |

Definition of abbreviations: “SNP” = single nucleotide polymorphism; “Chr.” = chromosome (1-22); “Chr. Location.” = chromosomal position of listed SNP; “β” = effect size (mL).

**^‡^**Liptak-combined P values for LOCCS and LODO.
